# *Missense Pathogenic variants in KIF4A* Affect Dental Morphogenesis Resulting in X-linked Taurodontism, Microdontia and Dens-Invaginatus

**DOI:** 10.3389/fgene.2019.00800

**Published:** 2019-09-20

**Authors:** Lord J.J. Gowans, Sophia Cameron-Christie, Rebecca L. Slayton, Tamara Busch, Miguel Romero-Bustillos, Steven Eliason, Mason Sweat, Nara Sobreira, Wenjie Yu, Piranit N. Kantaputra, Elizabeth Wohler, Wasiu Lanre Adeyemo, Salil A. Lachke, Deepti Anand, Collen Campbell, Bernadette K. Drummond, David M. Markie, W. Jansen van Vuuren, L. Jansen van Vuuren, Paul S. Casamassimo, Ronald Ettinger, Arwa Owais, I. van Staden, Brad A. Amendt, Adebowale A. Adeyemo, Jeffrey C. Murray, Stephen P. Robertson, Azeez Butali

**Affiliations:** ^1^Department of Biochemistry and Biotechnology, Kwame Nkrumah University of Science and Technology, Kumasi, Ghana; ^2^Department of Women’s and Children’s Health, Dunedin School of Medicine, University of Otago, Dunedin, New Zealand; ^3^Department of Pediatric Dentistry, University of Washington, Seattle, WA, United States; ^4^Department of Oral Pathology, Radiology and Medicine, University of Iowa, Iowa City, IA, United States; ^5^Department of Anatomy, University of Iowa, Iowa City, IA, United States; ^6^Institute of Genetic Medicine, John Hopkins University, Baltimore, MD, United States; ^7^Center of Excellence in Medical Genetics Research, Chiang Mai University, Chiang Mai, Thailand; ^8^Division of Pediatric Dentistry, Department of Orthodontics and Pediatric Dentistry, Faculty of Dentistry, Chiang Mai University, Chiang Mai, Thailand; ^9^Department of Oral and Maxillofacial Surgery, University of Lagos, Lagos, Nigeria; ^10^Department of Biological Sciences, University of Delaware, Newark, DE, United States; ^11^Department of Internal Medicine, University of Iowa, Iowa City, IA, United States; ^12^Department of Oral Sciences, University of Otago, Dunedin, New Zealand; ^13^Department of Pathology, Dunedin School of Medicine, University of Otago, Dunedin, New Zealand; ^14^Department of Pediatric Dentistry, Ohio State University, Columbus, OH, United States; ^15^Department of Prosthodontics, University of Iowa, Iowa City, IA, United States; ^16^Department of Pediatric Dentistry, College of Dentistry, University of Iowa, Iowa City, IA, United States; ^17^National Human Genomic Research Institute, Bethesda, MD, United States; ^18^Department of Pediatrics University of Iowa, Iowa City, IA, United States

**Keywords:** exome sequencing, X-linked recessive, microdontia, taurodontism, dens invaginatus

## Abstract

The etiology of dental anomalies is multifactorial; and genetic and environmental factors that affect the dental lamina have been implicated. We investigated two families of European ancestry in which males were affected by taurodontism, microdontia and dens invaginatus. In both families, males were related to each other *via* unaffected females. A linkage analysis was conducted in a New Zealand family, followed by exome sequencing and focused analysis of the X-chromosome. In a US family, exome sequencing of the X-chromosome was followed by Sanger sequencing to conduct segregation analyses. We identified two independent missense variants in *KIF4A* that segregate in affected males and female carriers. The variant in a New Zealand family (p.Asp371His) predicts the substitution of a residue in the motor domain of the protein while the one in a US family (p.Arg771Lys) predicts the substitution of a residue in the domain that interacts with Protein Regulator of Cytokinesis 1 (PRC1). We demonstrated that the gene is expressed in the developing tooth bud during development, and that the p.Arg771Lys variant influences cell migration in an *in vitro* assay. These data implicate missense variations in *KIF4A* in a pathogenic mechanism that causes taurodontism, microdontia and dens invaginatus phenotypes.

## Introduction

Human primary tooth development starts at 6 weeks of gestation through the appearance of the dental lamina, a distinct morphological thickening of the oral ectoderm, whereas permanent dentition starts developing between 10 to 13 weeks of gestation (Ooë, 1981). As tooth development progresses, the dental lamina invaginates into the underlying mesenchyme to form the tooth placodes which later progressively develop into the bud-shaped, cap-like and bell-like structures of developing teeth. These developmental stages of the tooth are characterized by localized expression of several genes, including *Edar*, *p21*, *Fgf20* and *Dkk4* (Ooë, 1981; Balic and Thesleff, 2015). *BMP*, *EDA*, *WNT/β-catenin*, *FGF* and *Shh* signaling pathways spatio-temporally regulate tooth morphogenesis as well as replacement and delineate the timing of individual tooth formation, teeth size, shape and position.^2^ Mutations in genes within these pathways have been associated with various dental anomalies in humans. Dental anomalies, including anodontia, hypodontia and oligodontia, amelogenesis imperfecta, and dentinogenesis imperfecta, has been observed in individuals with mutations in genes such as *PAX9, AXIN2, EDA, EDAR, WNT10A, WNT10B, EDARADD, LTBP3, LRP6, GREM2, SMOC MSX1, AMBN and COL1A2* (Juuri and Balic, 2017; Lu et al., 2018; Kantaputra et al., 2018)-

Taurodontism is a rare developmental dental condition that largely affects the molar teeth and may be associated with hypodontia. In this condition, the crown of the molar tooth and pulp chamber are disproportionately and vertically longer than the roots. It can either be seen in isolation or as part of a syndrome and/or chromosomal aneuploidies, especially those with ectodermal defects (Puttalingaiah et al., 2014; Yang et al., 2015). Microdontia defined as smaller than normal teeth with shortened crowns (vertically or mesio-distally) and loss of contact areas between the teeth, may be either localized, in which case only a small set of teeth are affected, or generalized. Microdontia is a mild form of hypodontia and can be part of a syndrome or as a consequence of environmental insults during development (Van der waal and Van der kwast, 1988; Bargale and Kiran, 2011; Boyle, 2011). Dens invaginatus or dens invagination is a tooth developmental anomaly that results from either the dental papilla folding into the developing tooth or the entire enamel organ folding into the dental papilla. In both instances, this leads to the formation of a tooth within a tooth, hence the term dens invaginatus (Kantaputra and Gorlin, 1992; Holan, 1998; Eden et al., 2002). There has been a previous report of individuals with both dens invaginatus and taurodontism (McNamara et al., 1998).

The Kinesin family member 4A (*KIF4A*) gene encodes an adenosine triphosphate (ATP)-dependent microtubule-based motor protein that participates in the intracellular transport of membranous organelles (Martinez et al., 2008; Tipton et al., 2017). The *KIF4A* protein may also maintain the integrity of chromosomes during mitosis by participating in mitotic chromosomal positioning and bipolar spindle stabilization because they have been observed, in addition to other proteins such as condensin I, to be associated with the condensed arms of chromosomes (Voets et al., 2015).

Here we report that missense variants in *KIF4A* (OMIM gene ID: 300521) cause an X-linked recessive triad of taurodontism, microdontia and dens invaginatus in two unrelated families. One variant (p.Asp317His, from a New Zealand family) predicts the substitution of a residue in the motor domain of the protein while the other (p.Arg771Lys, from a US family) predicts the substitution of a residue in the domain that interacts with Protein Regulator of Cytokinesis 1 (*PRC1*). We demonstrate that the gene is expressed in the developing tooth bud during development, and one of the variants (p.Arg771Lys) reported here influences cell migration in an *in vitro* assay.

## Material and Methods

### Whole Exome Sequencing (WES) in Family 1

The consensus coding sequence (CCDS) exonic regions and flanking intronic regions totaling ∼51 Mb were captured using the Agilent SureSelect XT kit and paired end 100 bp reads were generated with the Illumina HiSeq2500 platform. We aligned each read to the human genome reference (GRCh37) with the Burrows-Wheeler Alignment (BWA) v.0.5.10-tpx (Li and Durbin, 2009). Local realignment around indels and base call quality score recalibration was performed with the Genome Analysis Toolkit (GATK) v.2.3-9-ge5ebf34 (McKenna et al., 2010). Variant filtering was done *via* the Variant Quality Score Recalibration (VQSR) method (DePristo et al., 2011). Using the PhenoDB Variant Analysis Tool of PhenoDB, we prioritized heterozygous, homozygous, compound heterozygous and hemizygous rare functional variants (missense, nonsense, splice site variants, and indels) shared by the proband and his affected maternal uncle (IV-5) (Sobreira et al., 2015). We excluded variants with an allele frequency (MAF) >0.01 in dbSNP 126, 129, and 131, the Exome Variant Server (release ESP6500SI-V2) (Lek et al., 2016) and 1000 Genomes Project.

### Linkage Analyses and WES in Family 2

Genotyping was performed using the Omni1-Quad platform (Illumina). Linkage was performed with Allegro 2.0 with a disease frequency modeled at 0.0001 and disease penetrance as 0.001 for females and 0.99 for males (Gudbjartsson et al., 2005). Next, whole exome sequencing was performed on affected individual IV-1 on the Illumina HiSeq 2000 platform, using the SeqCapEZ2 capture kit (Roche). FASTQ files were aligned, processed and called on a pipeline based on the GATK Best Practice (McKenna et al., 2010). Reads were aligned with BWA-MEM (Li and Durbin, 2009), with exonic target regions covered at an average 44x depth. GATK and GATK 3.7 was used for VCF analysis (McKenna et al., 2010). Individual variant calling was done using GATK’s Haplotypecaller followed by joint genotyping with Genotype Variant Call File (GVCFs). The called variants were then compared to WES from 723 in-house controls called under the same protocol as the proband WES. Variants were excluded if depth was 2 or fewer reads, if their QUAL score was ≤30, the quality by depth (QD) was ≤2.0.

### Expression of Kif4a

Briefly, formalin-fixed paraffin embedded tissue sections for the *in-situ* hybridization was prepared and 8 µm sagittal sections were prepared. Digoxigenin-labeled probe was prepared using the DIG RNA Labeling Kit (Roche # 11175025910) using a template generated by the primers Kif4-F:

5’-TCCAAGCTGCATCTTGTAGACCTCGC-3’andKif4-SP6-R:5’ ATTTAGGTGACACTATAGAGTTCAGCTGTCTGGGGATCAATAT-3’.

### Functional Effect of the Kif4a Variant

*Kif4a* clone (Harvard Medical School, DF/HCC DNA Resource Core clone ID HsCD00331381) was subjected to site directed mutagenesis to introduce the c.304G > A variant identified in family 1. Products were purified and wild-type and mutant *Kif4a* were cloned into pcDNA3.1 using standard ligation techniques and sequence-confirmed. Triplicate transfections of clones (4 µg) into HEPM cells were performed using polyethyleneimine (PEI) at a DNA : PEI ratio of 1:3 and cells were seeded into 60 mm dishes at 60% confluency and cultured in DMEM without fetal bovine serum or antibiotics. In 24 h post-transfection, cells were detached using trypsin and seeded at maximum confluency. Three to 6 h after seeding, and once adherent cells were rinsed with PBS, a scratch was performed using a 200 µl pipette tip. The cell fields were imaged (Nikon Eclipse TS100 microscope and NIS-Element F3.2 software) at 0, 12, and 24 h with images analyzed in Fiji, a plugin of ImageJ (Rueden et al., 2017). The distance between the borders of the scratch was measured at t = 0 h and the number of cells between the lines was counted at subsequent time points and the comparisons over time were evaluated using a t-test (α = 0.05) in Excel. The same set of experiments was also performed in mesenchymal dental papilla cells.

## Results

### Clinical Information

**Figure 1:**
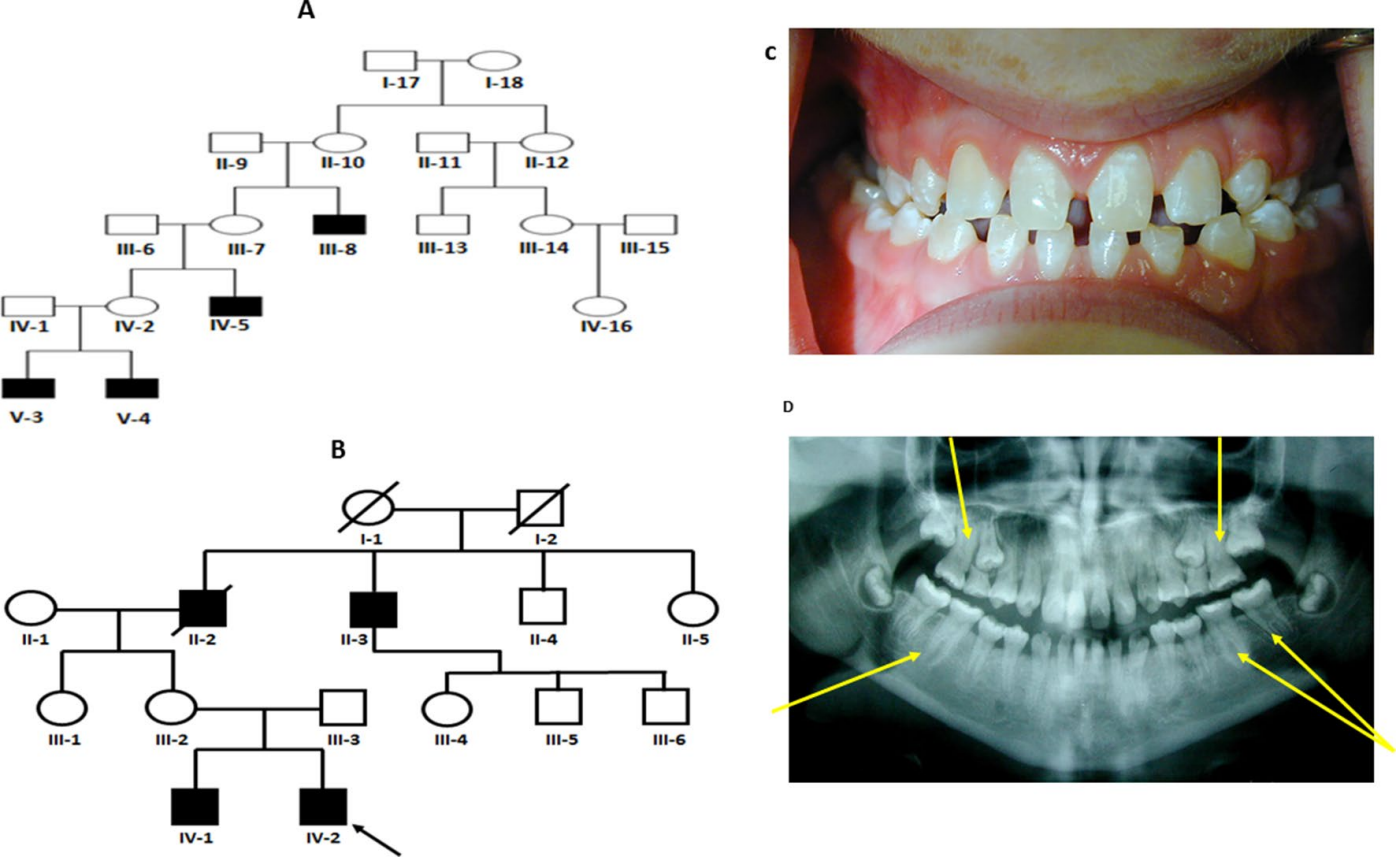
**(A** and **B)** Pedigree for family 1 and 2 showing affected males shaded in black. Individuals included in segregation analyses in the family are shown with black Panel **(C)** Frontal view from one twin 1 from family 1 showing generalized microdontia and spacing. Panel **(D)** is a panoramic radiograph from the same twin as above. He is in the late mixed dentition, as his maxillary second premolars have not yet erupted. Taurodontism in the molars is evident (arrows).

Two non-consanguineous families, who were independently evaluated in two separate centers, presented with a phenotype that is restricted to males with obligate carrier females exhibiting no clinical abnormalities (**Figure 1** and **Supplementary Figures 1**, **2**). Family 1, of European ancestry, has previously been described by Casamassimo et al in 1978 (Casamassimo et al., 1978).The affected child (V-3) his twin brother (V-4), their mother (IV-2) and uncle (IV-5) were clinically examined at the College of Dentistry Clinics at the University of Iowa, Iowa (**Table 1**). The phenotype, a combination of taurodontism, microdontia and dens invaginatus, as described previously, closely resembled that observed in the second family (**Figure 1A**). Family 2 was recruited in New Zealand (**Figure 1B**). Two siblings of European ancestry (IV-1 and IV-2) presented with a triad of taurodontism, microdontia and dens invaginatus, with a maternal grand-uncle (II-3) related through a reportedly affected maternal grandfather (II-2), reported to have exhibited an identical phenotype. The microdontia was generalized, the dens invaginatus affected canines and molars, and the molars were taurodont. There was decreased lower facial height and a flat facial profile. The dentition exhibited increased spacing with hypomineralized enamel and exposed dentine. These observations, in two unrelated and extensive pedigrees, of a male-limited phenotype transmitted exclusively through the matrilineal line and exhibiting no instances of male-to-male transmission led to the conclusion that this syndrome was an X-linked recessive disorder.

**Table 1:** Clinical features of affected individuals in family 1 and 2.

Family 1	Pedigree ID	Clinical features on examination	Clinical features from history
Child	V-3	Microdontia, dens invaginatus and taurodontism Straight profile Pulp calcifications Bilateral posterior crossbite Maxillary constriction	Normal neurodevelopment Kinky hair Normal height and weight
Twin brother	V-4	Microdontia, dens invaginatus and taurodontism Straight profile Pulp calcifications Bilateral posterior crossbite Maxillary constriction Over retained mandibular primary canines	
Maternal uncle	IV-5	Microdontia, dens invaginatus and taurodontism Pulp calcifications Posterior crossbite Impacted mandibular left first permanent premolar	Normal neurodevelopment
Maternal grand uncle	III.8	Microdontia, dens invaginatus and taurodontism	NA

**Family 2**			
Child 1	IV-1	Microdontia, dens invaginatus and taurodontism Decreased facial height Flat facial profile Hypomineralized enamel Exposed dentine	Normal neurodevelopment, developing some lower joint problems, dyslexia height - 10^th^ percentile weight - 25^th^ percentile
Child 2	IV-2		Normal neurodevelopment, hearing impairment height - 10^th^ percentile weight - 25^th^ percentile

### Variants in KIF4A Are Associated With Dental Triads in the Two Families

To begin the search for the genetic basis for this condition we first carried out whole exome sequencing of the X-chromosome on affected individuals from family 1. The proband (numbered V-4 in **Figure 1A**) and his maternal uncle (numbered IV-5 in **Figure 1A**) were sequenced and the data analyzed. Analysis of the WES datasets in Family 1, obtained from the affected proband and his affected maternal granduncle, identified two shared rare (allele frequency 0.005 in the 1000 Genomes Project, dbSNP build 131, Exome Variant Server release ESP6500SI-V2, or ExAC database) X chromosomal hemizygous variants: c.2312G > A (p.Arg771Lys) in exon 21 of *KIF4A* (NM_012310.4), and c.304G > A (p.Asp102Asn) in exon 3 of *GLOD5* (NM_001080489) (**Table 2**). These variants were both validated by Sanger sequencing. Polyphen-2 predicts that the p.Arg771Lys variant in *KIF4A* is probably damaging with a score of 0.998 whereas SIFT predicts that the variant is tolerated. For the p.Asp102Asn variant in *GLOD5*, Polyphen-2 predicted it to be damaging with a score of 1.000 and similarly SIFT also predicted that the variant was damaging.

**Table 2:** Genotypes of *KIF4A* and *GLOD5* variants obtained through Sanger Sequencing for available members of family.

Individual ID	Affected status	*KIF4A* genotypes for Arg771Lys variant at ChrX:69,615,600^a,b^	*GLOD5* genotypes for Asp102 Asn variant at ChX:48,629,445 ^a,b^
3	affected male proband	A	A
2	unaffected female	AG	AG
4	affected male twin	A	A
5	affected male	A	A
7	unaffected female	AG	AG
12	unaffected female	AG	GG
13	unaffected male	G	G
14	unaffected female	GG	GG
16	unaffected female	GG	GG

^a^Reference allele G and ^b^alternative allele A.

Since both pedigrees strongly imply that an X-linked basis for taurodontism, microdontia and dens invaginatus is a consistent triad phenotype, we further analyzed the segregation of the *KIF4A* and *GLOD5* variants in other members of Family 1 from whom we had available DNA (**Table 1**). Only the *KIF4A* variant segregated with the dental phenotype in this family, with key obligate female carriers (individuals IV-2, III.7 and female *KIF4A* variant carrier (individual III.12; **Figure 1A**) lacking the *GLOD5* variant **Table 2**.

To refine localization for the presumptive causative X-linked variant underlying this condition in Family 2 (**Figure 1B**), linkage was first performed using SNP array data for both affected brothers (IV-1, IV-2), together with their mother (III-2) and affected maternal grand-uncle (II-3). From the linkage analysis performed in family 2, two peaks on the X-chromosome, both with a maximum LOD score of 1.2 (**Supplementary Figure 3**) were observed. The first was 10.3 cm (between *rs12011068* and *rs6623997*, chrX:67,376,320-86,623,256, hg37) and the second was 4.8 cm (between *rs1569752* and *rs5978053*, chrX:128,058,708-134,013,529, hg37). Having defined these candidate regions, WES obtained on individual IV-1was then examined in Family 2. The following filtering criteria were applied: variants found on the X chromosome, must be rare (not present in either in-house controls, or the public datasets ExAC and EVS) and the variant PhastCons score must be <0.5 (Siepel et al., 2005). Only one variant met these criteria, a missense variant, c.949G > C in exon 8 of *KIF4A*. This variant predicts the substitution p.Asp317His and has a PhastCons score of 1.0, indicating high conservation at this position in vertebrates (**Supplementary Figures 4** and **5**). The substitution is predicted to lie within the motor domain of the protein. *KIF4A* maps to Xq13 extending from chrX:69,509,879-69,640,774 (hg37), within the interval mapped as being shared between all three affected individuals in this family (II-3, IV-1, IV-2; **Supplementary Figure 1**). PolyPhen2 also predicts this variant to be possibly damaging (score 0.918) and SIFT predicts it to be damaging.

It is possible that rather than being X-linked, a causal variant may be present on an autosome and obligate carrier females are explained by the incomplete-penetrance for the trait. To exclude this possibility the autosomal fraction of the sequenced exome should be examined for likely pathogenic variants. In family 2 we excluded any variants of any zygosity if they were present in either in-house controls or the public dataset ExAC. We then excluded variants where manual inspection of the reads with Integrative Genomics Viewer (IGV) indicated a likelihood of a sequencing artefact. This strategy defined a list of 50 variants, which was reduced to 21 if only variants with high or moderate protein-altering effect (frameshift, splice-site variant, novel stop codon, missense or in-frame indel) were considered. No autosomal genes containing variants were common between this list and the list generated from a similar autosomal analysis on Family 1.

The pedigree-based evidence for an X-linked, recessive etiology underlying this condition in both families, together with the co-segregation of missense pathogenic variants substituting highly conserved residues in *KIF4A* in the context of the exclusionary evidence shown by the segregation analysis in Family 1 and the linkage study in Family 2, constitutes compelling evidence for the role of these variants in *KIF4A* in the causation of this condition.

### Kif4a Is Expressed in the Developing Tooth Buds

**Figure 2:**
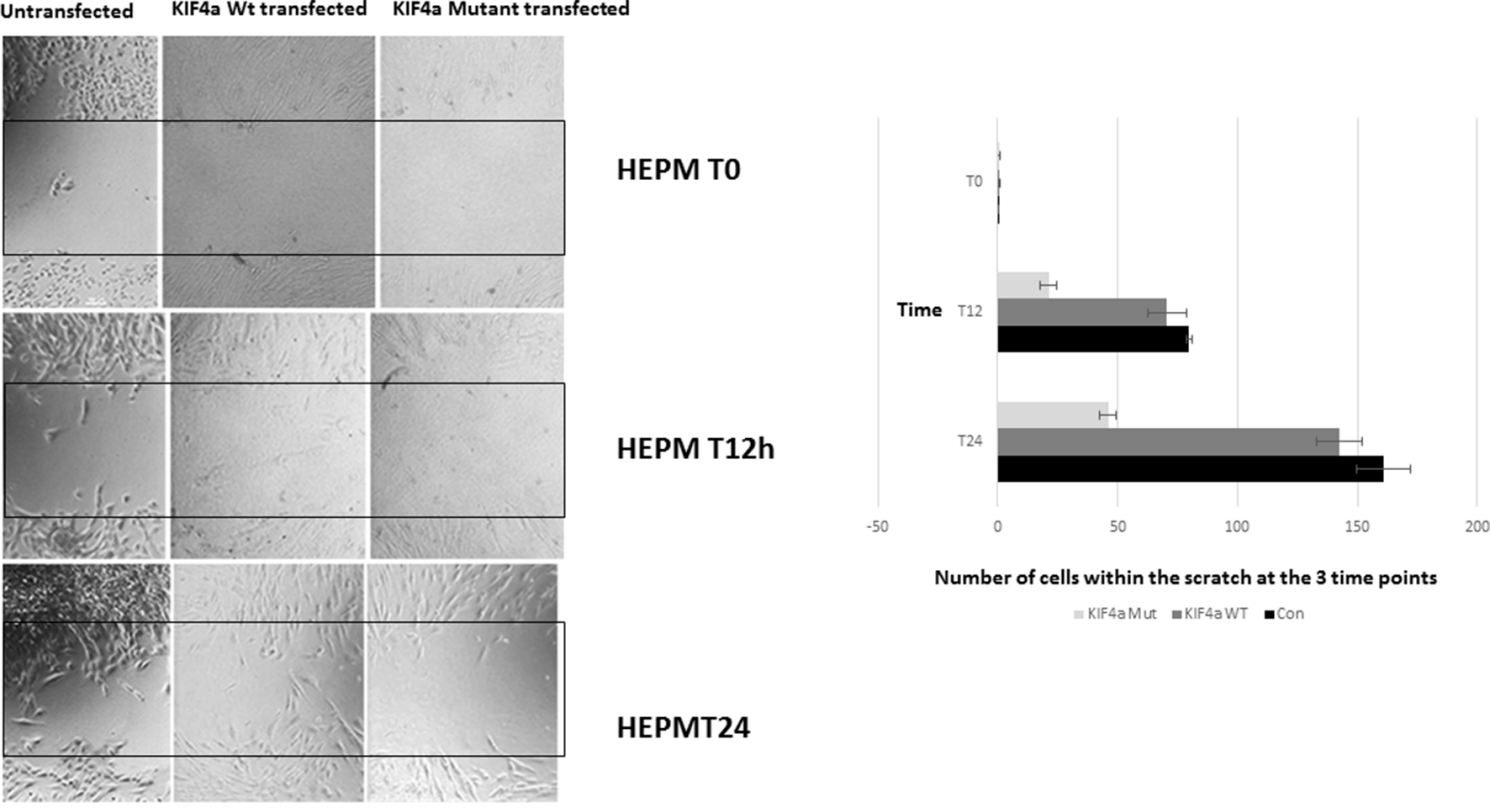
Migration scratch assay. HEPM cells were seeded at high density 24 h after Kif4α Wild-type & Kif4α Mutant transfection. Pictures at 0, 4, 8, 20, and 30 h after scratch. The number of cells between the red lines were counted in the different groups. Box plots of the number of cells in the scratched area at the different time points.

These new observations of *Kif4a* variants being associated with a dental phenotype prompted us to further examine the expression of this gene during development. Using the systems tool for tooth expression-based gene discovery - a bioinformatics tool for identifying the expression pattern of genes using microarray data - we investigated the expression pattern of *Kif4* (*Kif4* is mouse ortholog of *KIF4A* in human) in the developing tooth at different time points in mouse tooth development by analyzing whole-genome microarray expression datasets on isolated dental mesenchyme and epithelium (GSE32321) at embryonic (E) stages E10.0, E11.0, E11.5, E12.0, E12.5, E13.0, E13.5 and E14.5 that were generated on Illumina WG- 6 platform. Further, we analyzed mouse isolated tooth tissue at E13.5 (GSE32334), E14.0 (GSE27429) and P10 (GSE7164) generated on the Affymetrix Mouse 430 2.0 platform. Normalized gene expression fluorescent signal intensities were computed using ‘affy’ and ‘lumi’ packages by importing datasets in the ‘R’ statistical environment as described previously (Gautier et al., 2004; Du et al., 2008; Anand et al., 2015). We sought to further extend these observations through *in situ* hybridization, using previously reported protocols (Gregorieff and Clevers, 2015; Sun et al., 2016). The systems tool for tooth expression-based gene discovery showed that *Kif4a* is expressed in the developing tooth buds in embryonic mice at E15, consistent with this newly established role in tooth morphogenesis (**Supplementary Figure 6**). We observed that *Kif4a* is expressed in the mesenchyme of the lower incisors, tongue, mandible and maxilla (**Figure 2**).

### Kif4a Mutant Delays Migration of the Cells

**Figure 3:**
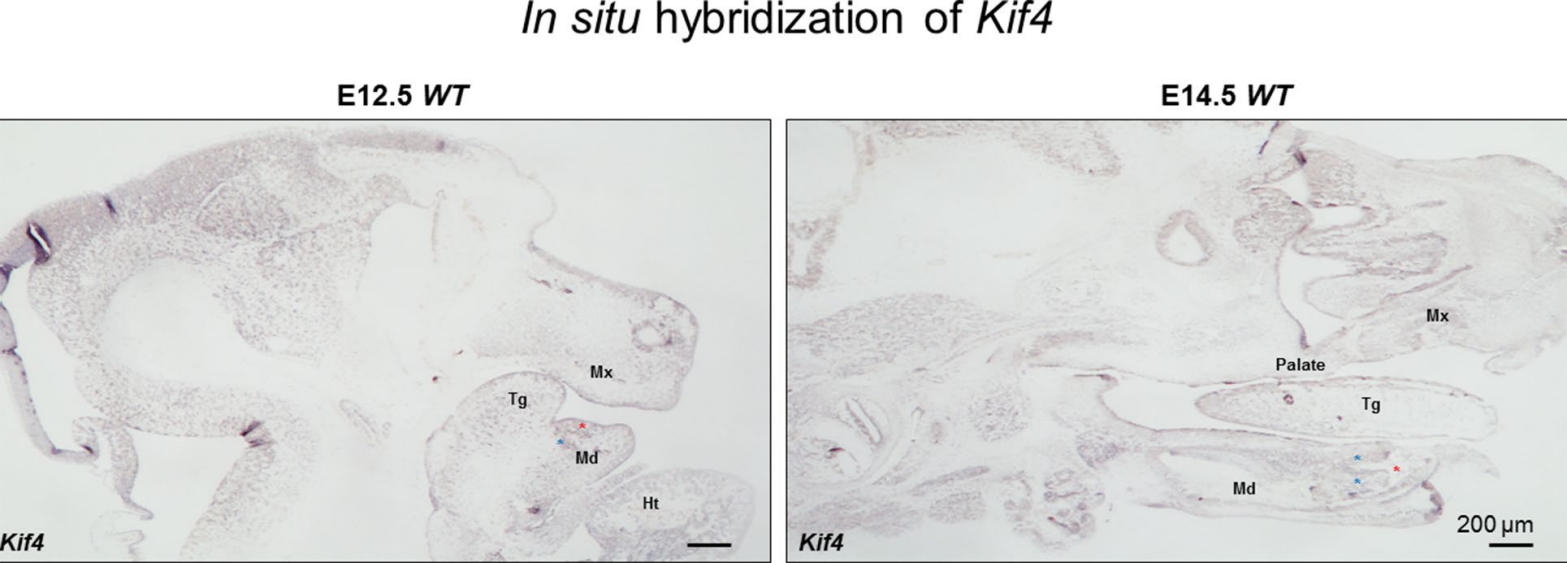
The in situ hybridization of Kif4 in E12.5 and E14.5 embryos. Red asterisks show lower incisors, blue asterisks show mesenchyme adjacent the lower incisors. Tg, tongue; Md, mandible; Mx, maxilla; Ht, heart. Scale bar: 200 μm.

To evaluate if the *KIF4A* variant found in family 1 conferred a functional effect on the protein, we assessed its effect on cell proliferation and migration as previously described for *KIF4A* (Bieling et al., 2010). We also examined the expression of the KIF4A using western blot and confirmed over-expression of KIF4A (**Supplementary Figure 7**). A migration scratch assay was performed in Human Embryonic Palatal Mesenchymal (HEPM) cells and mesenchymal dental papilla cells (**Figure 3**). In this assay, after the performance of a scratch across a monolayer of cultured HEPM cells, the appearance of cells within the scratch area over time can be measured as a proxy for cell migration/proliferation. Notably, HEPM cells do not migrate in a cell sheet covering the scratched area; instead, isolated cells migrate to the middle of the scratched area at different time points. We did not observe any statistically significant alteration in the number of cells found in the scratched area between *Kif4a* wild-type and the mutant at 0 h (p = .0.58). However, we observed a significant difference between *Kif4a* wild-type and mutant at 12 h (p = 0.003). This was more obvious at 24 h (p = 0.0002). When we compared untransfected cells and mutant cells we observed significant differences at 12 h (p = 4×10^-5^) and 24 h (p = 3.3 × 10^-5^), respectively. Finally, there was no difference between the untransfected cells and *Kif4a* wild-type in all the time points; 0 h (p = 0.47), 12 h (p = 0.91) and 24 h (p = 0.06).

## Discussion

We observed two independent missense variants (p.Asp317His and p.Arg771Lys) in *KIF4A* in individuals with a phenotype of taurodontism, microdontia and dens invaginatus segregating in males from two unrelated families. Variants in *KIF4A* leading to exon-skipping and missense pathogenic variants have been reported in individuals with intellectual disability (ID) (Willemsen et al., 2014). It is unclear whether the Dutch ID pathogenic variant is fundamentally different in terms of the pathophysiology of the disease mechanism relating to the dental phenotype reported here. Our scratch test experiments indicate that one of our variants does affect cell behavior, but further investigation is needed to understand why there is no effect on cognitive function in affected individuals in our study and to determine if the phenotypes are mutation specific.

The *KIF4A* protein may also be essential for the organization of the central spindle prior to cytokinesis by translocating the protein regulator of cytokinesis 1 (*PRC1*) and co-localizing with the chromosomal passenger complex (CPC) and central spindling at the plus ends of inter-digitating spindle microtubules during the metaphase to anaphase transition, a crucial process for the formation of an organized central spindle midzone and midbody, and for successful cytokinesis Subramanian et al., 2010; Abadía-Molina et al., 2017). Aurora B phosphorylates *KIF4A*, inducing microtubule-dependent ATPase activity of *KIF4A* as well as promoting its interaction with *PRC1* (Nunes Bastos et al., 2013). The *PRC1-*interaction domain is a critical domain for the activity of *KIF4A* and missense variants such as p.Arg771Lys we observed in this domain may affect *KIF4A* interaction and cytokinesis. The p.Asp317His variant located in the kinesin motor domain of KIF4A introduces a neutral charge compared to the wild type protein and may disturb the motor domain and abolish its function (Protein structure analysis of mutations causing inheritable diseases, 2010).

Dens invaginatus are the defects of abnormal invagination of Hertwig epithelial root sheath cells and the epithelial cells of the enamel organ, respectively. It is possible that the variants (p.Asp317His and p.Arg771Lys) we observed may lead to dysregulation of epithelial invagination. Additional studies to confirm this is needed. Late timing or failure of Hertwig’s epithelial root sheath (HERS) invagination may be responsible for the apical shift of the root furcation in taurodontism (Yang et al., 2015). Pathogenic variants in genes in the Eda receptor ligand pair, particularly Eda and Edar, may result in taurodontism of some molar teeth as a result of changes in the proliferation and angle of HERS (Fons Romero et al., 2017).

The data we present here demonstrates that missense variants in *KIF4A*, a gene that is located on the X chromosome, are implicated in a pathogenic mechanism that also produces taurodontism, microdontia and dens invaginatus phenotypes. Thus, we have demonstrated that variants in *KIF4A* cause X-linked recessive taurodontism, microdontia and dens invaginatus.

## Ethics Statement

This study was carried out in accordance with the recommendation of Human subject research guidelines and Human subject research committee at the University of Iowa and University of Otago with written informed consent from all subjects. All subjects gave written informed consent in accordance with the Declaration of Helsinki. The protocol was approved by the University of Iowa and University of Otago IRB.

## Author Contributions

LG performed the Sanger sequencing and wrote initial draft. SC-C did the linkage analyses and exome sequencing and revised the manuscript draft. RS, AO, PC and RE screened family 1, collected the saliva for DNA, and contributed to the manuscript. TB coordinated DNA handling for family 1 and Sanger sequencing and contributed to the manuscript. BD clinically evaluated family 2, collected the saliva for DNA, and contributed to the manuscript. MR-B, SE, MS, and WY did the *in situ*, scratch experiments and contributed to the manuscript. NS and EW did the exome sequencing for family 1 and analyzed and contributed to the manuscript. FK, WLA, CC, DM, WV, IVS, AA, and JM contributed to the analyses and data interpretation as well as manuscript development and revision. SL and DA did the bioinformatics analyses for expression of KIF4A in tooth tissues and contributed the final draft of the manuscript. AB and SR conceived the project, supervised the analyses, and drafted the manuscript. LV clinically evaluated family 2, collected the saliva for DNA, and contributed to the manuscript. BA designed and supervised the in situ, scratch experiments and contributed to the manuscript.

## Funding

This work was supported by Curekids New Zealand (SC-C, SR), NIDCR K99/R00 Grant DE022378 and Robert Wood Johnson Foundation Grant number 72429 (AB), R90 DE024296 (MR-B), R37 grants DE-08559 and DE-016148 (JM), Ghana Cleft Foundation, G001 (LG), The Center of Excellence in Medical Genetics Research, Chiang Mai University; the Thailand Research Fund; The Dental Association of Thailand; and The Faculty of Dentistry, Chiang Mai University (PK) and National Human Genome Research Institute grant 5U54HG006542 (AAA).

## Conflict of Interest Statement

The authors declare that the research was conducted in the absence of any commercial or financial relationships that could be construed as a potential conflict of interest.
