# Supplementary figures and images for: Missense Pathogenic variants in KIF4A Affect Dental Morphogenesis Resulting in X-linked Taurodontism, Microdontia and Dens-Invaginatus
Source: Front Genet. 2019 Sep 20;10:800. doi: 10.3389/fgene.2019.00800 (PMC6764483; doi:10.3389/fgene.2019.00800)

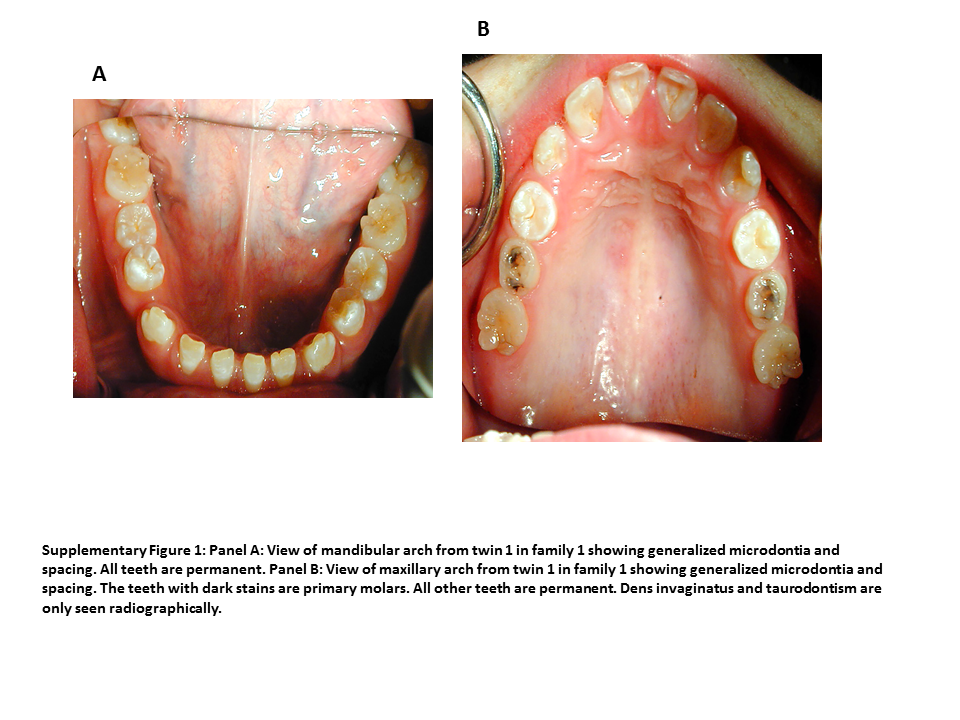

Supplement: Supplementary file 1 [file Image_1.tif]

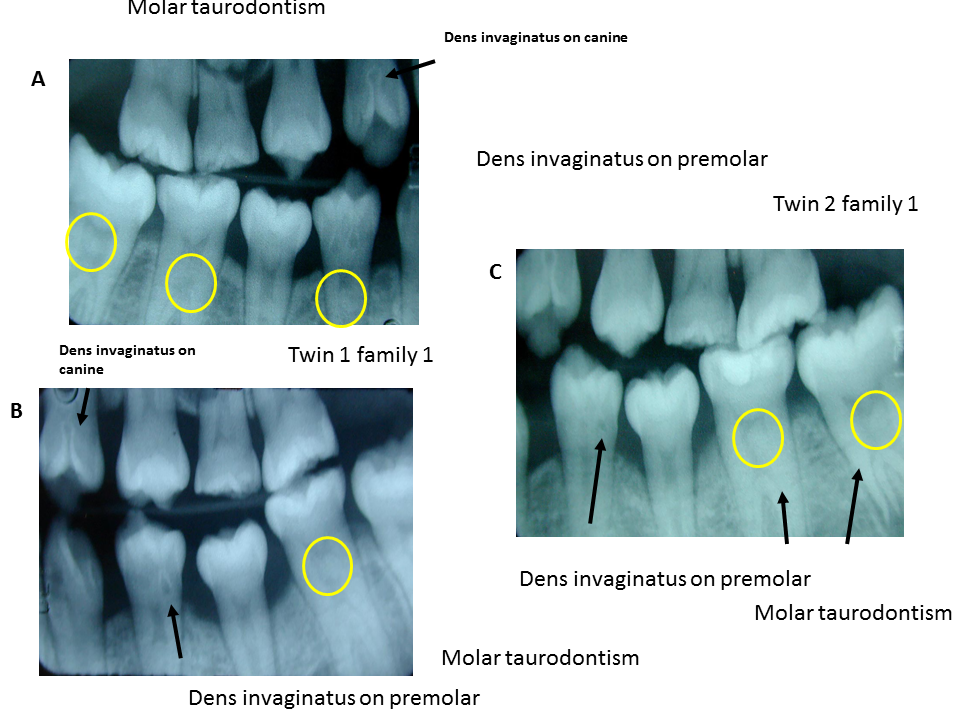

Supplement: Supplementary file 2 [file Image_2.tif]

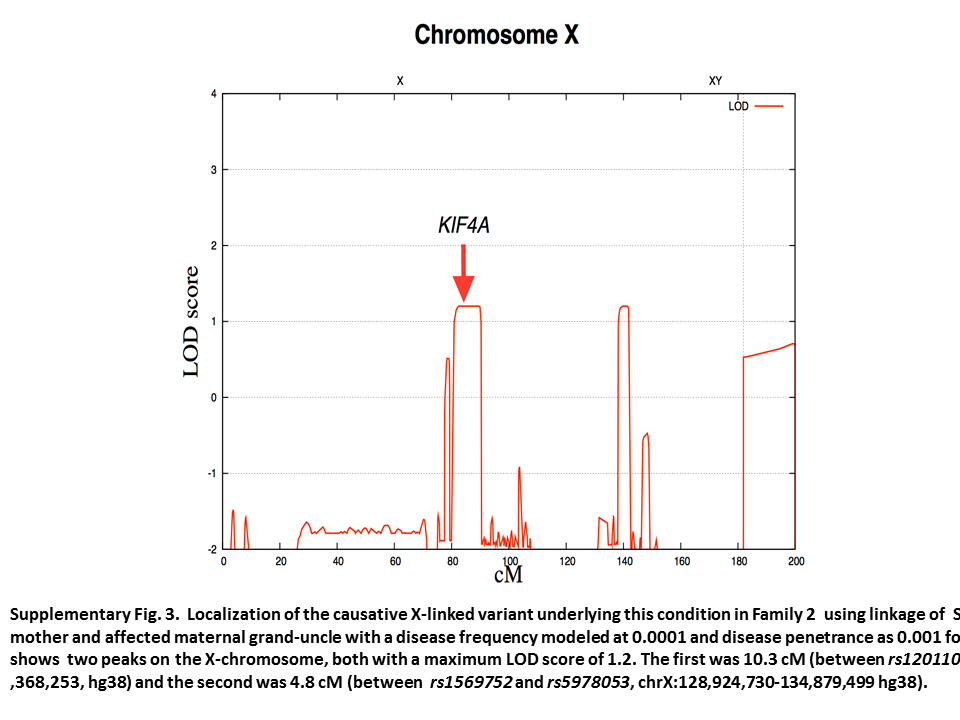

Supplement: Supplementary file 3 [file Image_3.tif]

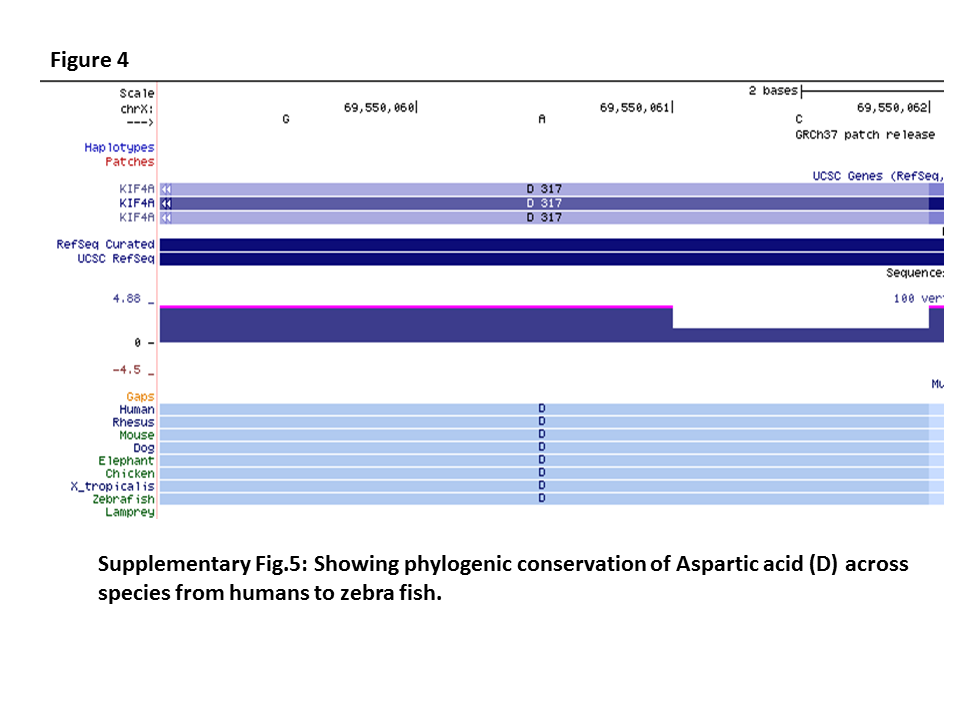

Supplement: Supplementary file 4 [file Image_4.tif]

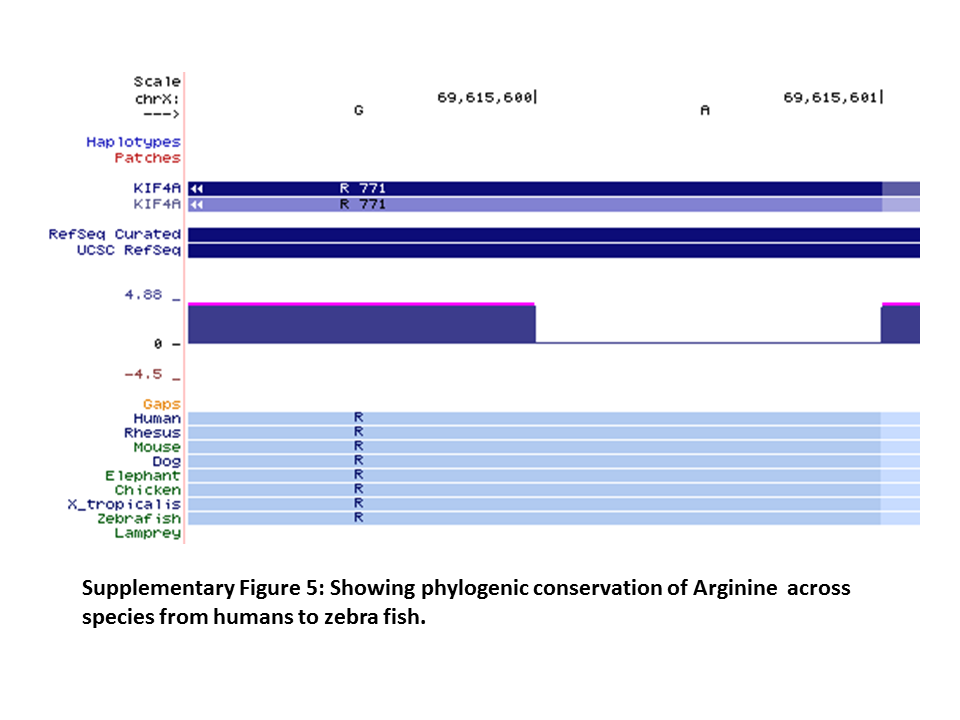

Supplement: Supplementary file 5 [file Image_5.tif]

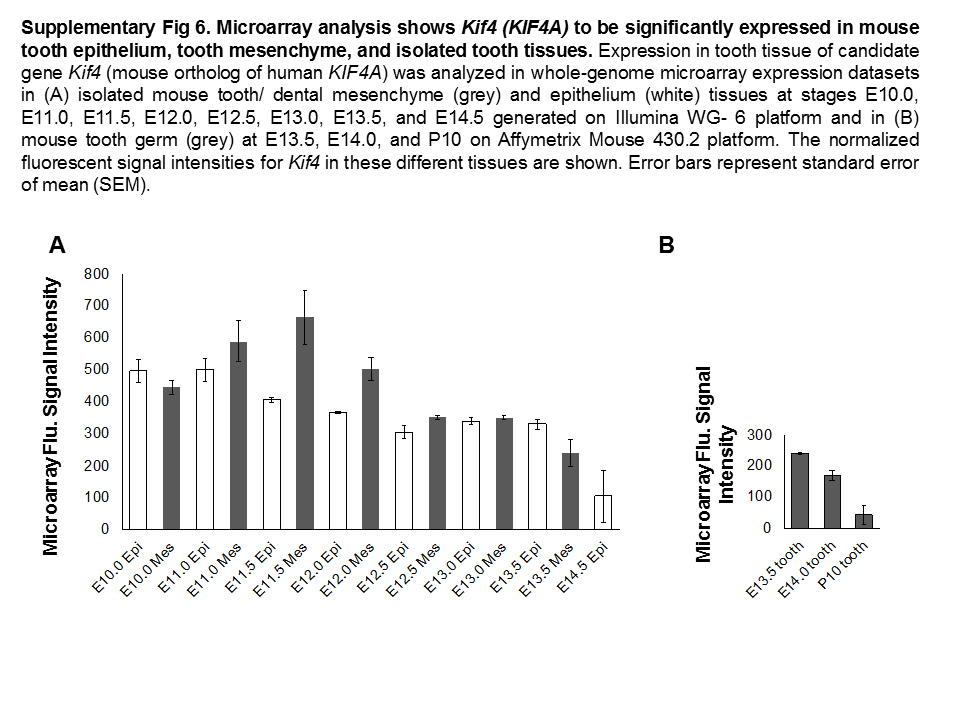

Supplement: Supplementary file 6 [file Image_6.tif]

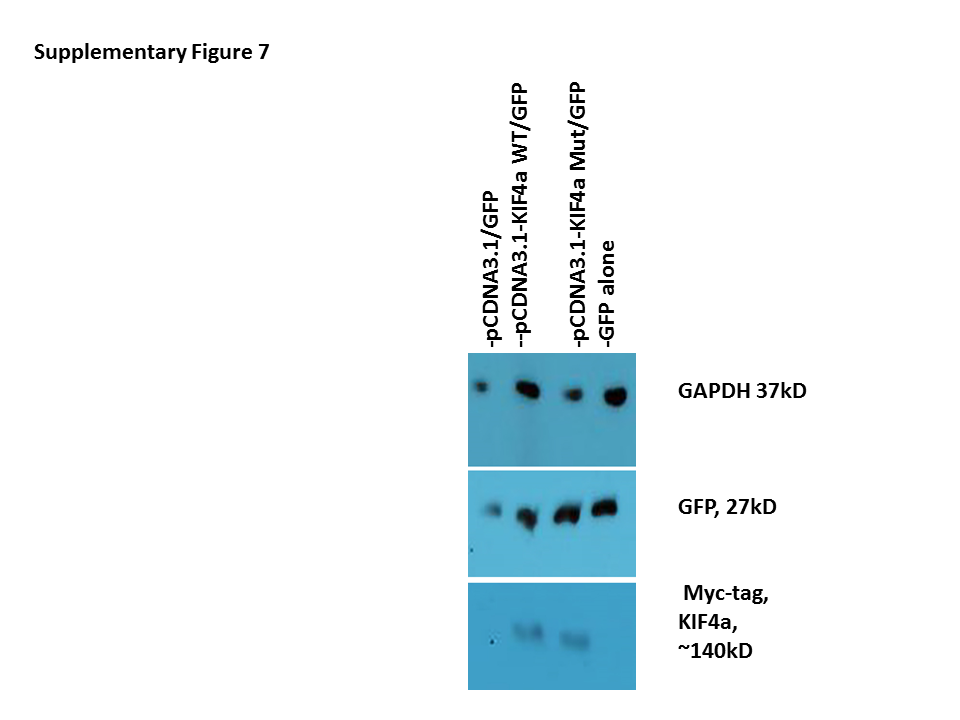

Supplement: Supplementary file 7 [file Image_7.tif]
